# Long-term Incidence Rates of Esophageal Squamous Cell Carcinoma in Chinese Patients With Low-grade Intraepithelial Neoplasia and Association of Surveillance Endoscopy With Incidence

**DOI:** 10.1001/jamanetworkopen.2022.47415

**Published:** 2022-12-19

**Authors:** He Li, Hongliang Wu, Maomao Cao, Yiwen Yu, Jinyi Zhou, Shaokai Zhang, Feng Tong, Jiyong Gong, Huadong Wang, Fan Yang, Siyi He, Xinxin Yan, Shaoli Zhang, Pengfei Luo, Hengmin Ma, Ling Liang, Changfa Xia, Wanqing Chen

**Affiliations:** 1 Office of Cancer Screening, National Cancer Center/National Clinical Research Center for Cancer/Cancer Hospital, Chinese Academy of Medical Sciences and Peking Union Medical College/Chinese Academy of Medical Sciences Key Laboratory for National Cancer Big Data Analysis and Implementation, Beijing, China; 2 Department of Anesthesiology, National Cancer Center/National Clinical Research Center for Cancer/Cancer Hospital, Chinese Academy of Medical Sciences and Peking Union Medical College, Beijing, China; 3 Department for Chronic Non-communicable Diseases Prevention and Control, Jiangsu Provincial Center for Disease Control and Prevention (Public Health Research Institute of Jiangsu Province), Nanjing, China; 4 Department of Cancer Epidemiology, Affiliated Cancer Hospital of Zhengzhou University/Henan Cancer Hospital, Henan Engineering Research Center of Cancer Prevention and Control, Henan International Joint Laboratory of Cancer Prevention, Zhengzhou, China; 5 Department of Preventive Management, Shandong Cancer Hospital and Institute, Shandong First Medical University and Shandong Academy of Medical Sciences, Jinan, China; 6 Institute of Chronic Non-communicable Diseases Prevention and Control, Anhui Provincial Center for Disease Control and Prevention, Hefei, China

## Abstract

**Importance:**

Surveillance endoscopy is recommended for patients with low-grade intraepithelial neoplasia (LGIN); high-quality evidence about the use of surveillance endoscopy and esophageal squamous cell carcinoma (ESCC) incidence in patients with LGIN is important but limited.

**Objective:**

To estimate long-term ESCC incidence rates in patients with LGIN and the association between surveillance endoscopy and ESCC incidence.

**Design, Setting, and Participants:**

This community-based, multicenter, prospective cohort study in 9 regions in rural China included patients with LGIN diagnosed by endoscopic screening between July 1, 2007, and December 31, 2016; all participants were followed up until December 31, 2021.

**Main Outcomes and Measures:**

The primary outcome was ESCC incidence. The ESCC standardized incidence ratio (SIR) was estimated using sex- and age-specific incidence in the general population of rural China in 2010 and hazard ratios (HRs) and 95% CIs were calculated using Cox proportional hazards models.

**Results:**

A total of 3258 patients with LGIN were included; 1772 (54.39%) were men, with a mean (SD) age of 58.21 (6.97) years. Among them, 1378 patients (42.30%) underwent at least 1 surveillance endoscopy (surveillance group) and 1880 (57.70%) did not undergo any surveillance endoscopy (nonsurveillance group). During the follow-up period (median, 7.96 years; IQR, 6.08-10.54 years), 170 ESCC cases were diagnosed, with a cumulative incidence of 6.28 per 1000 person-years. A higher incidence of ESCC (incidence rate, 7.07 per 1000 person-years) was observed in the nonsurveillance group than in the surveillance group (incidence rate, 5.14 per 1000 person-years). Patients with LGIN in the surveillance group had a lower SIR (SIR, 4.07; 95% CI, 1.13-10.34) than those in the nonsurveillance group (SIR, 5.65; 95% CI, 2.00-12.58); however, patients with LGIN in both groups had a higher risk of ESCC than the general population. Patients in the surveillance group had a 31% decreased risk of ESCC incidence (HR, 0.69; 95% CI, 0.50-0.95) compared with those in the nonsurveillance group, after adjusting for baseline risk factors.

**Conclusions and Relevance:**

In this prospective cohort study, patients with LGIN had a higher risk of developing ESCC than the general population, and endoscopic surveillance was associated with a decrease in ESCC incidence in these patients.

## Key Points

**Question:**

What is the incidence of esophageal squamous cell carcinoma (ESCC) in patients with low-grade intraepithelial neoplasia (LGIN) and is there an association between surveillance endoscopy and ESCC incidence?

**Findings:**

In this cohort study of 3258 patients with LGIN in China, the cumulative ESCC incidence was 6.28 per 1000 person-years during a median follow-up of 7.96 years. Patients in the surveillance group had a 31% decreased risk of ESCC incidence.

**Meaning:**

The findings of this study present insights into the efficiency of surveillance endoscopy for patients with LGIN in the Chinese population, thereby providing evidence for surveillance guidelines for ESCC.

## Teaser

This cohort study examines the incidence of esophageal squamous cell carcinoma (ESCC) and the use of surveillance endoscopy in patients with low-grade intraepithelial neoplasia in China.

## Introduction

Esophageal cancer (EC) is the seventh most common cancer and the sixth highest cause of cancer mortality worldwide.^1^ In 2020, there were an estimated 0.48 million esophageal squamous cell carcinoma (ESCC) cases worldwide, accounting for 84% of all EC cases. China accounts for nearly half of the ESCC disease burden worldwide, and patients with ESCC have a poor prognosis with a population-based 5-year survival rate of 30.3%.^2,3^ The prognosis of ESCC is closely related to the tumor stage at diagnosis. A multicenter, hospital-based cohort study on patients with EC in China reported 5-year survival rates ranging from 61.90% for stage I to 26.17% for stage IV EC.^4^ Endoscopic screening provides an opportunity for the early detection of ESCC or its precursors, which helps reduce ESCC-associated incidence and mortality.^5,6^

Esophageal mild dysplasia or moderate dysplasia, namely, low-grade intraepithelial neoplasia (LGIN), is the precursor lesion for ESCC,^7^ and patients with LGIN are recommended for endoscopic surveillance in the guidelines for ESCC screening and early detection and early treatment (EDET) in China.^8^ This surveillance strategy has also been applied in the National Key Public Health Projects in China, namely, the screening and EDET program in the high-risk areas of EC in rural China, the Esophageal, Stomach, and Liver Cancer Screening Program (ESLCSP), and the Cancer Screening Program in urban China. These 3 programs provide endoscopic screening and regular surveillance endoscopy for patients with LGIN diagnosed at baseline screening.^9^ The primary aim of endoscopic surveillance in patients with LGIN is to reduce new EC-related cases and deaths by detecting and treating premalignant lesions and early-stage asymptomatic EC. To our knowledge, the existing studies in patients with LGIN estimated progression risk based on populations only in high-risk areas in China,^10,11,12,13^ and the risk of developing EC in patients with LGIN relative to that in the general population has not been reported. In addition, no population-based cohort study has examined the use of surveillance endoscopy for preventing EC. The lack of solid evidence on surveillance endoscopy in patients with LGIN has limited the quality of evidence of surveillance endoscopy recommendations in the guidelines for ESCC screening and EDET in China.

We conducted a community-based, multicenter, prospective cohort study based on the ESLCSP to estimate long-term ESCC incidence rates among individuals with LGIN and the use of endoscopic surveillance and ESCC incidence. The findings may provide evidence for guidelines regarding the use of surveillance endoscopy for preventing ESCC.

## Methods

### Study Design and Participants

In this prospective cohort study, participants in the ESCC screening and EDET program of the ESLCSP were enrolled. The study design of this program has been described in detail previously.^14^ Participants enrolled in the present study were required to meet the following inclusion criteria: a baseline endoscopy with pathologic diagnosis of LGIN from the ESLCSP in China between January 2007 and December 2016. The exclusion criteria were as follows: (1) patients with cancer that was diagnosed before enrollment, (2) participants younger than 40 years or older than 69 years at baseline, and (3) participants with incomplete baseline information. The study population was from 9 rural regions of 4 provinces in China, including 3 regions (Hongze, Jinhu, and Yandu) in Jiangsu Province, 3 regions (Wenshang, Tengzhou, and Mudan) in Shandong Province, 2 regions (Xiangfu and Yuzhou) in Henan Province, and 1 region (Panji) in Anhui Province. The study protocol was independently approved by the institutional review board of the Cancer Institute and Hospital, Chinese Academy of Medical Sciences. Participants provided written informed consent; no financial compensation was provided. This study followed the Strengthening the Reporting of Observational Studies in Epidemiology (STROBE) reporting guideline for cohort studies.

### Baseline Procedure and Data Collection

In the included centers, residents aged 40 to 69 years without a self-reported cancer history were approached through personal contact and telephone invitation by trained local medical staff. All eligible participants were administered a baseline questionnaire by trained staff to gather information, including age at enrollment, sex, socioeconomic status, smoking status, alcohol consumption, and family history of cancer and cancer type. Anthropometric measurements were obtained for each individual at baseline, including height, weight, heart rate, and blood pressure. These variables were used for the initial assessment to identify high-risk individuals. Detailed descriptions and classification standards for each evaluated variable and the estimated effectiveness of this initial screening strategy have been previously described in detail.^14^

High-risk individuals identified by the initial assessment were recommended to undergo standard upper gastrointestinal endoscopic examination and biopsy with iodine staining. The protocol of endoscopic examination and pathologic diagnosis in the ESCC screening and EDET program was formulated based on the official endoscopy protocol of EDET.^15^ Endoscopists in each study center were trained on the protocol by experts from the Chinese Academy of Medical Sciences and Peking Union Medical College. The participants were given a local anesthetic (5 mL of a slurry of lidocaine, 1%-2%) orally before the procedure. The entire esophagus was visually examined, and the esophagus was sprayed with 10 to 15 mL of aqueous iodine, 1.2% (Lugol iodine solution), which stains normal mucosa brown and leaves dysplastic lesions unstained.^16,17^ Biopsies were collected from suspicious lesions; the number of biopsies depended on the size of the lesion. Biopsies were fixed in formaldehyde, 10% to 13%, embedded in paraffin, and stained with hematoxylin and eosin. Two experienced pathologists independently reviewed the biopsy slides, and diagnostic discrepancies were adjudicated by consultation. Based on the histologic criteria from the official EDET protocol, the biopsies were categorized into the following diagnoses: normal, acanthosis, atrophy, basal cell hyperplasia, esophagitis, mild dysplasia, moderate dysplasia, severe dysplasia, carcinoma in situ, intramucosal carcinoma, submucosal carcinoma, and invasive carcinoma.

### Outcome and Surveillance Procedures

The primary outcome was ESCC incidence. In our ESCC screening and EDET program, endoscopic surveillance has been provided since 2012. This surveillance strategy was as follows: patients with mild dysplasia were recommended to undergo triennial surveillance endoscopy and those with moderate dysplasia were recommended to undergo annual surveillance endoscopy. All patients with LGIN diagnosed at the baseline endoscopy in 2007-2011 were invited to undergo endoscopic reexamination in 2012 and were recommended to undergo subsequent surveillance endoscopy in accordance with the surveillance strategy. Patients with LGIN diagnosed at the baseline endoscopy since 2012 were invited to receive endoscopic surveillance according to the surveillance strategy. During endoscopic surveillance, we continued to provide endoscopic surveillance for patients with LGIN who did not follow the recommended surveillance interval to lower the ESCC incidence as much as possible. The procedures and protocols of endoscopic examination and biopsy during surveillance endoscopy were the same as those used at baseline. In addition, endoscopists were informed of the location of any lesions identified at baseline to ensure a careful review of the endoscopic images.

Patients with severe dysplasia or carcinoma in situ diagnosed at the surveillance endoscopy and all ESCC were followed up to collect tumor-related information (pathologic type and stage) and treatment-related information, including endoscopic mucosal resection, endoscopic submucosal dissection, multi-band mucosectomy, radiofrequency ablation, surgery, chemotherapy, and radiotherapy.

### Statistical Analysis

Participants were divided into 2 groups: those who did not undergo surveillance endoscopy (nonsurveillance group) and those who had undergone at least 1 surveillance endoscopy (surveillance group). Continuous variables are presented as the mean (SD) or median (IQR), and categorical variables are reported as counts and percentages. We compared baseline characteristics in these 2 groups using χ^2^ tests for categorical variables and *t* tests for continuous variables. All statistical tests were 2 sided, and *P* < .05 was considered statistically significant. Person-years of follow-up in each group were calculated from the date of enrollment to the date of diagnosis of ESCC or December 31, 2021, whichever occurred first. Cumulative incidence rates in each group were calculated as the number of cancer cases divided by the person-years of follow-up. We calculated the expected numbers of ESCC cases by multiplying the observed sex- and age-specific (5-year age group) person-years at risk in the study population by the corresponding incidence in the general population of rural China in 2010.^18^ We report the observed to expected cases as a standardized incidence ratio (SIR); the 95% CIs assumed an exact Poisson distribution.^19^ Cox proportional hazards regression models were used to estimate hazard ratios (HRs) and 95% CIs for associations between endoscopic surveillance and the risk of ESCC incidence. In addition, 2 sensitivity analyses were conducted to reassess the outcomes of surveillance in reducing the ESCC incidence. First, we reanalyzed the multivariate HR of endoscopic surveillance and ESCC incidence by conducting multiple imputations for missing variables (using 10 imputations combined using Rubin rules). Second, we excluded participants enrolled before 2012 and reanalyzed the multivariate HR. Analyses were performed using SAS software, version 9.4 (SAS Institute Inc).

## Results

### Study Flow and Demographic Characteristics of the Analysis Cohort

In total, 3354 participants with LGIN diagnosed at baseline endoscopy for ESCC screening between July 1, 2007, and December 31, 2016, were eligible for this study. Of these, 96 individuals were excluded, and the remaining 3258 patients with LGIN were included in the final analysis. Among the 3258 patients with LGIN, 1772 (54.39%) were men and 1486 (45.61%) were women, with a mean (SD) age of 58.21 (6.97) years; 1378 individuals (42.30%) underwent at least 1 surveillance endoscopy, and 1880 (57.70%) did not undergo any surveillance endoscopy (Figure).

**Figure.**
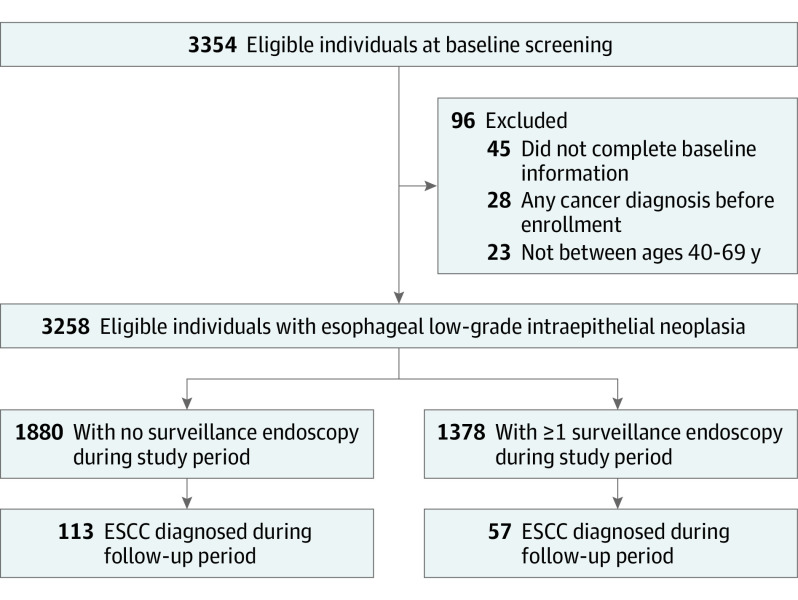
Flow Diagram of the Study Participants ESCC indicates esophageal squamous cell carcinoma.

The main demographic characteristics were compared between the surveillance and nonsurveillance groups. Compared with patients with LGIN who did not have a family history of EC, those with a family history of EC were more likely to undergo surveillance endoscopy. There were no significant group differences in sex, age, educational level, smoking status, alcohol consumption, or body mass index (Table 1).

**Table 1.** Baseline Characteristics of the Patients

Characteristic	No. (%)		*P* value
	Patients with no surveillance endoscopy	Patients with ≥1 surveillance endoscopy	
Total, No.	1880	1378	
Sex			
Male	1032 (54.89)	740 (53.70)	.50
Female	848 (45.11)	638 (46.30)	
Age			
Mean (SD), y	58.05 (7.14)	58.42 (6.74)	.13
Median (IQR), y	59 (53-64)	59 (54-64)	
Educational level			
≤Primary school	1237 (65.80)	903 (65.53)	.87
≥Middle school	643 (34.20)	475 (34.47)	
Study region, province			
Jiangsu	730 (38.83)	518 (37.59)	.89
Anhui	92 (4.89)	70 (5.08)	
Shandong	905 (48.14)	680 (49.35)	
Henan	153 (8.14)	110 (7.98)	
Family history of EC			
No	1626 (86.49)	1142 (82.87)	.004
Yes	254 (13.51)	236 (17.13)	
Cigarette smoking			
No	1229 (65.37)	891 (64.66)	.67
Yes	651 (34.63)	487 (35.34)	
Alcohol use			
No	1326 (70.53)	951 (69.01)	.35
Yes	554 (29.47)	427 (30.99)	
BMI, mean (SD)	23.99 (2.58)	24.06 (2.68)	
No. of surveillance visits			
1	NR	1079 (78.30)	NR
2	NR	231 (16.76)	
≥3	NR	68 (4.94)	
Surveillance by recommended interval			
No	NR	934 (67.78)	NR
Yes	NR	444 (32.22)	

Abbreviations: BMI, body mass index (calculated as weight in kilograms divided by height in meters squared); EC, esophageal cancer; NR, not relevant.

### Risk of ESCC in Patients With LGIN

The median follow-up time was 7.96 years (IQR, 6.08-10.54 years) in the 3258 patients with LGIN, which accounted for a total of 27 081.26 person-years. During the follow-up period, ESCC was diagnosed in 170 individuals in the study population, with a cumulative incidence of 6.28 per 1000 person-years. Among these patients, 113 ESCC cases occurred in the nonsurveillance group (incidence rate, 7.07 per 1000 person-years), and there were 57 ESCC cases in the surveillance group (incidence rate, 5.14 per 1000 person-years). Higher incidence rates of ESCC were observed in men, individuals older than 50 years, those with a lower educational level, and those with a family history of EC (Table 2).

**Table 2.** Incidence Rates of ESCC

Variable	Total			No surveillance endoscopy			≥1 Surveillance endoscopy		
	Person-years	Observed ESCC cases	Incidence rate per 1000 person-years	Person-years	Observed ESCC cases	Incidence rate per 1000 person-years	Person-years	Observed ESCC cases	Incidence rate per 1000 person-years
Total	27 081.26	170	6.28	15 992.97	113	7.07	11 088.29	57	5.14
Sex									
Male	14 461.28	107	7.40	8673.82	74	8.53	5787.45	33	5.70
Female	12 619.98	63	4.99	7319.15	39	5.33	5300.84	24	4.53
Age, y									
40-49	4042.47	13	3.22	2547.11	11	4.32	1495.36	2	1.34
50-59	10 479.61	59	5.63	6261.54	38	6.07	4218.07	21	4.98
60-69	12 559.18	98	7.80	7184.32	64	8.91	5374.86	34	6.33
Educational level									
≤Primary school	17 891.56	118	6.60	10 543.86	78	7.40	7347.70	40	5.44
≥Middle school	9189.70	52	5.66	5449.11	35	6.42	3740.59	17	4.54
Family history of EC									
No	22 850.05	134	5.86	13 845.03	93	6.72	9005.02	41	4.55
Yes	4231.21	36	8.51	2147.94	20	9.31	2083.27	16	7.68

Abbreviations: EC, esophageal cancer; ESCC, esophageal squamous cell carcinoma.

Table 3 reports the SIR of ESCC in the study population. Compared with the general population, patients with LGIN had a significantly increased risk of ESCC, with an SIR of 5.00 (95% CI, 1.62-11.67). Although patients in the surveillance group had a lower SIR (SIR, 4.07; 95% CI, 1.13-10.34) than those in the nonsurveillance group (SIR, 5.65; 95% CI, 2.00-12.58), they both had a higher risk of ESCC than the general population. Subgroup analyses showed that, compared with the general population, a higher risk of ESCC existed in women (SIR, 6.00; 95% CI, 2.20-13.06), those with a lower educational level (SIR, 4.44; 95% CI, 1.32-10.87), and those with a family history of EC (SIR, 5.33; 95% CI, 1.81-12.13) among patients of LGIN in the surveillance group. Patients with LGIN in the nonsurveillance group, regardless of sex, educational level, and family history of EC, had a higher risk of ESCC than the general population.

**Table 3.** SIR of ESCC

Variable	Total cohort			No surveillance endoscopy			≥1 Surveillance endoscopy		
	Expected ESCC cases	Observed ESCC cases	SIR (95% CI)	Expected ESCC cases	Observed ESCC cases	SIR (95% CI)	Expected ESCC cases	Observed ESCC cases	SIR (95% CI)
Total	34	170	5.00 (1.62-11.67)	20	113	5.65 (2.00-12.58)	14	57	4.07 (1.13-10.34)
Sex									
Male	24	107	4.46 (1.33-10.90)	15	74	4.93 (1.58-11.57)	10	33	3.30 (0.75-9.22)
Female	9	63	7.00 (2.81-14.42)	5	39	7.80 (3.32-15.50)	4	24	6.00 (2.20-13.06)
Educational level									
≤Primary school	22	118	5.36 (1.83-12.17)	13	78	6.00 (2.20-13.06)	9	40	4.44 (1.32-10.87)
≥Middle school	12	52	4.33 (1.26-10.72)	7	35	5.00 (1.62-11.67)	5	17	3.40 (0.80-9.36)
Family history of EC									
No	28	134	4.79 (1.51-11.37)	17	93	5.47 (1.89-12.33)	11	41	3.73 (0.96-9.85)
Yes	5	36	7.20 (2.94-14.69)	3	20	6.67 (2.61-13.98)	3	16	5.33 (1.81-12.13)

Abbreviations: EC, esophageal cancer; ESCC, esophageal squamous cell carcinoma; SIR, standardized incidence ratio.

### Outcomes of Surveillance Endoscopy

Among 26 ESCC cases with the specific tumor stage, 21 (80.77%) were detected at an early stage (stage 0/I/II) in the surveillance group, and this percentage was much higher than that in the nonsurveillance group (49.12% [28 of 57]) (eTable 1 in Supplement 1). Patients with ESCC in the surveillance group more frequently underwent an endoscopic resection (endoscopic submucosal dissection or endoscopic mucosal resection) compared with those in the nonsurveillance group (eTable 1 in Supplement 1). In addition, among 56 patients with severe dysplasia or carcinoma in situ detected at surveillance endoscopy, 78.57% (n = 44) underwent an endoscopic resection treatment (eTable 2 in Supplement 1); patients who underwent treatment had a significantly lower risk of developing ESCC than those refusing treatment (HR, 0.12; 95% CI, 0.02-0.89).

In patients who underwent at least 1 surveillance endoscopy, a significantly decreased ESCC risk was observed (HR, 0.72; 95% CI, 0.52-0.98; *P* = .04). After adjusting for baseline risk factors (sex, age, educational level, smoking status, and alcohol consumption), patients in the surveillance group had a 31% decreased risk of developing ESCC (HR, 0.69; 95% CI, 0.50-0.95) compared with those in the nonsurveillance group (Table 4). In addition, compared with patients with LGIN in the nonsurveillance group, those undergoing surveillance endoscopy within the recommended interval had a 38% lower risk of developing ESCC (HR, 0.62; 95% CI, 0.42-0.92); this significantly lower risk of developing ESCC was not observed among those undergoing surveillance endoscopy outside of the recommended interval (eTable 3 in Supplement 1).

**Table 4.** Adjusted Risk of Progression to ESCC in Patients With Low-grade Intraepithelial Neoplasia Over a Median Follow-up Period of 7.96 Years

Variable	Individuals, No. (%)	Person years	No. of ESCC Cases	Univariate HR (95% CI)	*P* value	Multivariable HR (95% CI)	*P* value
Surveillance endoscopy							
No	1880 (57.70)	15 992.97	113	1 [Reference]	NA	1 [Reference]	NA
Yes	1378 (42.30)	11 088.29	57	0.72 (0.52-0.98)	.04	0.69 (0.50-0.95)	.02
Sex							
Male	1772 (54.39)	14 461.28	107	1 [Reference]	NA	1 [Reference]	NA
Female	1486 (45.61)	12 619.98	63	0.68 (0.50-0.93)	.02	0.73 (0.48-1.10)	.13
Age, y							
40-49	448 (13.75)	4042.47	13	1 [Reference]	NA	1 [Reference]	NA
50-59	1223 (37.54)	10 479.61	59	1.71 (0.94-3.12)	.08	1.65 (0.90-3.03)	.10
60-69	1587 (48.71)	12 559.18	98	2.32 (1.30-4.13)	.005	2.24 (1.24-4.04)	.01
Educational level							
≤Primary school	2140 (65.68)	17 891.56	118	1 [Reference]	NA	1 [Reference]	NA
≥Middle school	1118 (34.32)	9189.70	52	0.86 (0.62-1.20)	.38	0.86 (0.60-1.22)	.39
Family history of EC							
No	2768 (84.96)	22 850.05	134	1 [Reference]	NA	1 [Reference]	NA
Yes	490 (15.04)	4231.21	36	1.45 (1.00-2.10)	.05	1.50 (1.03-2.17)	.03
Cigarette smoking							
No	2120 (65.07)	17 610.82	98	1 [Reference]	NA	1 [Reference]	NA
Yes	1138 (34.93)	9470.44	72	1.37 (1.01-1.86)	.04	1.07 (0.72-1.60)	.73
Alcohol use							
No	2277 (69.89)	18 977.32	108	1 [Reference]	NA	1 [Reference]	NA
Yes	981 (30.11)	8103.94	62	1.35 (0.99-1.84)	.06	1.10 (0.74-1.65)	.63

Abbreviations: EC, esophageal cancer; ESCC, esophageal squamous cell carcinoma; HR, hazard ratio; NA, not applicable.

The sensitivity analysis (eTable 4 in Supplement 1) showed that patients with LGIN in the surveillance group had a decreased risk of developing ESCC compared with the nonsurveillance group. This decreased risk was noted even after multiple imputations were conducted (n = 3303; HR, 0.69; 95% CI, 0.62-0.76) and participants who had enrolled before 2012 (n = 2071; HR, 0.60; 95% CI, 0.38-0.96) were excluded.

## Discussion

In this community-based, multicenter, prospective cohort study in the Chinese population with a median follow-up period of 7.96 years, we found that patients diagnosed with LGIN remained at a higher risk of developing ESCC than the general population. In addition, surveillance endoscopy in patients with LGIN was associated with a reduced ESCC incidence compared with that in patients with no surveillance. This surveillance endoscopy association persisted even after adjusting for several demographic and lifestyle-related factors.

Patients with LGIN are recommended to undergo surveillance endoscopy, according to the current guidelines for ESCC screening and EDET in China.^8^ A prospective cohort study with a 13-year follow-up period of 682 participants from high-risk areas of ESCC in China who underwent endoscopic screening in the 1980s showed that patients with LGIN had an approximately 1.9 to 8.8 times higher risk of developing ESCC than healthy individuals.^10^ Recently, 2 additional community-based cohort studies conducted in high-risk areas also showed a higher risk of ESCC incidence in patients with LGIN than in healthy individuals.^12,13^ Consistent with these prospective studies, our findings also showed that patients with baseline LGIN have a higher risk of developing ESCC than the general population. In addition, among patients with LGIN who did not undergo surveillance endoscopy, each subpopulation stratified by sex, educational level, and family history of EC had a higher risk of ESCC than the general population. These findings add new high-quality evidence to support that surveillance endoscopy should be recommended to patients with LGIN and will provide fundamental support for the guidelines of ESCC screening and EDET in China.

The findings of this study emphasize the importance of surveillance endoscopy for patients with LGIN. First, the large benefit of endoscopic surveillance among patients with LGIN is demonstrated by the improved proportion of early-stage ESCC cases identified as well as lesions noted at the severe dysplasia or carcinoma in situ stage, which improved the proportion of patients who underwent curative endoscopic resection treatment, compared with those who did not undergo surveillance endoscopy. Second, surveillance endoscopy in patients with LGIN was associated with a 31% reduction in ESCC risk after adjusting for several demographic and lifestyle-related factors. This association was also noted in the sensitivity analysis that conducted multiple imputations and excluded participants enrolled before 2012. Our findings show that family history of EC and age were 2 independent factors for the development of ESCC. Therefore, patients with LGIN with a family history of EC and those older than 50 years should receive more attention during surveillance endoscopy.

### Strengths and Limitations

The primary strength of this study is that, to our knowledge, it is the first community-based, multicenter, prospective cohort study to estimate the outcome of surveillance endoscopy in patients with esophageal LGIN. Some findings, including the relative risk of ESCC in patients with LGIN compared with that of the general population and the use of surveillance endoscopy in preventing ESCC, provide evidence for the surveillance guideline of ESCC screening and EDET in China and in other countries that have an increased incidence of ESCC.

Our study has limitations. First, we did not collect detailed information on the potential variables in the endoscopic images, such as the size of the iodine-unstained lesions, so the confounders could not be assessed with greater precision.^20^ In our ongoing ESCC screening and surveillance program, we have collected sufficient information on endoscopic images since 2019. Therefore, a more comprehensive analysis integrating endoscopic images could be conducted in the future. Second, ESCC was the most common (>85%) pathologic subtype for EC in the Chinese population; however, to our knowledge, the sex- and age-specific incidences of ESCC in the Chinese population have not been reported. Therefore, the SIR of ESCC in our study was calculated by the incidence of EC in the general population of China in 2010, which would be underestimated. Under these circumstances, patients with LGIN still had a higher risk of developing ESCC than the general population, which suggests that these patients need regular surveillance to lower the ESCC incidence. Third, the completeness of the tumor stage and treatment data was approximately 50%, primarily because patients with cancer tended to receive examinations and treatments in large cities outside their home counties. We tried to obtain the stage and treatment data by contacting the patients or their relatives and locating the records in local cancer registries and hospitals; however, these data were unavailable for more than 50% of the patients. Recently, the National Cancer Center of China implemented a series of measures, such as improving the National Cancer Hospitals’ network, which would enhance the data quality of the ESCC screening and surveillance program. More detailed data on the tumor stages and treatment will be obtained in the future, and further analyses of cancer stage and cancer mortality will be conducted. Fourth, the current conclusions were based on a median follow-up of 7.96 years; longer-term follow-up is needed to substantiate our findings, especially in the subgroup that did not undergo surveillance endoscopy.

## Conclusions

In this prospective cohort study of patients with LGIN, our findings support surveillance endoscopy for patients with LGIN, as they have a higher risk of developing ESCC than the general population. In addition, surveillance endoscopy in patients with LGIN is associated with a reduction in ESCC incidence. In the future, we will continue to follow up the surveillance cohort and provide updates and key information, such as the tumor stages and treatment, detailed endoscopic images, and survival rates.

## Notes

Supplement 1. **eTable 1.** Characteristics of Esophageal Squamous Cell Carcinoma (ESCC) Patients According to Surveillance Endoscopy **eTable 2.** Risk of Esophageal Squamous Cell Carcinoma (ESCC) in Patients With Severe Dysplasia/Carcinoma In Situ Diagnosed by Surveillance Endoscopy, Compared With Treatment (No or Yes) **eTable 3.** Adjusted Risk of Progression to Esophageal Squamous Cell Carcinoma (ESCC) in Patients With Low-grade Intraepithelial Neoplasia Over a Median Follow-up Period of 7.96 Years, Adjusted by Surveillance Endoscopy Visits and Surveillance Interval **eTable 4.** Sensitivity Analysis of the Association Between Surveillance Endoscopy and the Incidence of ESCC

Supplement 2.
Data Sharing Statement

## References

[1] Sung H, Ferlay J, Siegel RL, . Global Cancer Statistics 2020: GLOBOCAN estimates of incidence and mortality worldwide for 36 cancers in 185 countries. CA Cancer J Clin. 2021;71(3):209-249. doi:10.3322/caac.21660 33538338

[2] Arnold M, Ferlay J, van Berge Henegouwen MI, Soerjomataram I. Global burden of oesophageal and gastric cancer by histology and subsite in 2018. Gut. 2020;69(9):1564-1571. doi:10.1136/gutjnl-2020-321600 32606208

[3] Zeng H, Chen W, Zheng R, . Changing cancer survival in China during 2003-15: a pooled analysis of 17 population-based cancer registries. Lancet Glob Health. 2018;6(5):e555-e567. doi:10.1016/S2214-109X(18)30127-X 29653628

[4] He Y, Liang D, Du L, . Clinical characteristics and survival of 5283 esophageal cancer patients: a multicenter study from eighteen hospitals across six regions in China. Cancer Commun (Lond). 2020;40(10):531-544. doi:10.1002/cac2.12087 32845581 PMC7571391

[5] Wei WQ, Chen ZF, He YT, . Long-term follow-up of a community assignment, one-time endoscopic screening study of esophageal cancer in China. J Clin Oncol. 2015;33(17):1951-1957. doi:10.1200/JCO.2014.58.0423 25940715 PMC4881309

[6] Chen R, Liu Y, Song G, . Effectiveness of one-time endoscopic screening programme in prevention of upper gastrointestinal cancer in China: a multicentre population-based cohort study. Gut. 2021;70(2):251-260. 32241902 10.1136/gutjnl-2019-320200 PMC7815635

[7] Lin DC, Wang MR, Koeffler HP. Genomic and epigenomic aberrations in esophageal squamous cell carcinoma and implications for patients. Gastroenterology. 2018;154(2):374-389. doi:10.1053/j.gastro.2017.06.066 28757263 PMC5951382

[8] He J, Chen WQ, Li ZS, ; Expert Group of China Guideline for the Screening, Early Detection and Early Treatment of Esophageal Cancer; Work Group of China Guideline for the Screening, Early Detection and Early Treatment of Esophageal Cancer. China guideline for the screening, early detection and early treatment of esophageal cancer (2022, Beijing). Article in Chinese. Zhonghua Zhong Liu Za Zhi. 2022;44(6):491-522. 35754225 10.3760/cma.j.cn112152-20220517-00348

[9] Cao M, Li H, Sun D, . Cancer screening in China: the current status, challenges, and suggestions. Cancer Lett. 2021;506:120-127. doi:10.1016/j.canlet.2021.02.017 33684533

[10] Wang GQ, Abnet CC, Shen Q, . Histological precursors of oesophageal squamous cell carcinoma: results from a 13 year prospective follow up study in a high risk population. Gut. 2005;54(2):187-192. doi:10.1136/gut.2004.046631 15647178 PMC1774842

[11] Wen D, Zhang L, Wang X, . A 5.5-year surveillance of esophageal and gastric cardia precursors after a population-based screening in China. J Gastroenterol Hepatol. 2015;30(12):1720-1725. doi:10.1111/jgh.13040 26183370

[12] Wei WQ, Hao CQ, Guan CT, . Esophageal histological precursor lesions and subsequent 8.5-year cancer risk in a population-based prospective study in China. Am J Gastroenterol. 2020;115(7):1036-1044. doi:10.14309/ajg.0000000000000640 32618654 PMC7477846

[13] Liu M, Zhou R, Guo C, . Size of Lugol-unstained lesions as a predictor for risk of progression in premalignant lesions of the esophagus. Gastrointest Endosc. 2021;93(5):1065-1073.e3. doi:10.1016/j.gie.2020.09.020 32950597

[14] Chen W, Li H, Zheng R, . An initial screening strategy based on epidemiologic information in esophageal cancer screening: a prospective evaluation in a community-based cancer screening cohort in rural China. Gastrointest Endosc. 2021;93(1):110-118.e2. doi:10.1016/j.gie.2020.05.052 32504698

[15] Detection CE; Treatment Expert Group. Bureau of Disease Control, Ministry of Health. Technology Scheme for Cancer Early Detection and Treatment. People’s Medical Publishing House; 2011.

[16] Dawsey SM, Fleischer DE, Wang GQ, . Mucosal iodine staining improves endoscopic visualization of squamous dysplasia and squamous cell carcinoma of the esophagus in Linxian, China. Cancer. 1998;83(2):220-231. doi:10.1002/(SICI)1097-0142(19980715)83:2<220::AID-CNCR4>3.0.CO;2-U 9669803

[17] Muto M, Minashi K, Yano T, . Early detection of superficial squamous cell carcinoma in the head and neck region and esophagus by narrow band imaging: a multicenter randomized controlled trial. J Clin Oncol. 2010;28(9):1566-1572. doi:10.1200/JCO.2009.25.4680 20177025 PMC2849774

[18] Chen W, Zheng R, Zhang S, . Esophageal cancer incidence and mortality in China, 2010. Thorac Cancer. 2014;5(4):343-348. doi:10.1111/1759-7714.12100 26767022 PMC4704347

[19] Breslow NE, Day NE. Statistical methods in cancer research: volume II—the design and analysis of cohort studies. IARC Sci Publ. 1987;(82):1-406. 3329634

[20] Liu M, Liu Z, Liu F, . Absence of iodine staining associates with progression of esophageal lesions in a prospective endoscopic surveillance study in China. Clin Gastroenterol Hepatol. 2020;18(7):1626-1635.e7. doi:10.1016/j.cgh.2019.08.058 31518715

