# Supplementary material for: Long-term Incidence Rates of Esophageal Squamous Cell Carcinoma in Chinese Patients With Low-grade Intraepithelial Neoplasia and Association of Surveillance Endoscopy With Incidence
Source: JAMA Netw Open. 2022 Dec 19;5(12):e2247415. doi: 10.1001/jamanetworkopen.2022.47415 (PMC9856485; doi:10.1001/jamanetworkopen.2022.47415)
Supplement: Supplement 1. — eTable 1. Characteristics of Esophageal Squamous Cell Carcinoma (ESCC) Patients According to Surveillance Endoscopy eTable 2. Risk of Esophageal Squamous Cell Carcinoma (ESCC) in Patients With Severe Dysplasia/Carcinoma In Situ Diagnosed by Surveillance Endoscopy, Compared With Treatment (No or Yes) eTable 3. Adjusted Risk of Progression to Esophageal Squamous Cell Carcinoma (ESCC) in Patients With Low-grade Intraepithelial Neoplasia Over a Median Follow-up Period of 7.96 Years, Adjusted by Surveillance Endoscopy Visits and Surveillance Interval eTable 4. Sensitivity Analysis of the Association Between Surveillance Endoscopy and the Incidence of ESCC [file jamanetwopen-e2247415-s001.pdf]

Supplementary Online Content

Li H, Wu H, Cao M, et al. Long-term incidence rates of esophageal squamous cell carcinoma in Chinese patients with low-grade intraepithelial neoplasia and association of surveillance endoscopy with incidence. *JAMA Netw Open*. 2022;5(12):e2247415. doi:10.1001/jamanetworkopen.2022.47415

eTable 1. Characteristics of Esophageal Squamous Cell Carcinoma (ESCC) Patients According to Surveillance Endoscopy
eTable 2. Risk of Esophageal Squamous Cell Carcinoma (ESCC) in Patients With Severe Dysplasia/Carcinoma In Situ Diagnosed by Surveillance Endoscopy, Compared With Treatment (No or Yes)
eTable 3. Adjusted Risk of Progression to Esophageal Squamous Cell Carcinoma (ESCC) in Patients With Low-grade Intraepithelial Neoplasia Over a Median Follow-up Period of 7.96 Years, Adjusted by Surveillance Endoscopy Visits and Surveillance Interval
eTable 4. Sensitivity Analysis of the Association Between Surveillance Endoscopy and the Incidence of ESCC

This supplementary material has been provided by the authors to give readers additional information about their work.

© 2022 Li H et al. *JAMA Network Open.*

eTable 1. Characteristics of esophageal squamous cell carcinoma (ESCC) patients according to surveillance endoscopy

**Characteristics**	ESCC patients in the non-surveillance group, n (%)	ESCC patients in the surveillance group, n (%)	P value
**Gender**			0.33
Male	74 (65.49)	33 (57.89)	
Female	39 (34.51)	24 (42.11)	
**Age at diagnosis, years**			<0.001
mean ± SD	62.80 (7.02)	64.59 (5.85)	
**Tumor stage***			0.007
0/I/II	28 (49.12)	21 (80.77)	
III/IV	29 (50.88)	5 (19.23)	
**Treatment†**			0.007
No treatment	6 (9.52)	2 (5.56)	
ESD/EMR	9 (14.29)	16 (44.44)	
Surgery	22 (34.93)	11 (30.55)	
Chemotherapy/Radiotherapy	13 (20.63)	1 (2.78)	
Surgery+ Chemotherapy/Radiotherapy	13 (20.63)	6 (16.67)	

Note: * 87 ESCC cases without information on histological stage: 56 in the non-surveillance group and 31 in the surveillance group. †71 ESCC cases without information on treatment: 50 in the non-surveillance group and 21 in the surveillance group. Abbreviations: EMR, endoscopic mucosal resection; ESCC, esophageal squamous cell carcinoma; ESD, endoscopic submucosal dissection.

© 2022 Li H et al. *JAMA Network Open.*

eTable 2. Risk of esophageal squamous cell carcinoma (ESCC) in patients with severe dysplasia/ carcinoma in situ diagnosed by surveillance endoscopy, compared with treatment (no or yes)

Treatment	No. of patients of severe dysplasia/ carcinoma in situ diagnosed by surveillance	No. of ESCC cases	Incidence rate per 1000 PYs	Univariate HR (95% CI)	Multivariable HR* (95% CI)
No	12	5	49.79		
Yes	44	3	7.39	0.15 (0.04-0.63)	0.12 (0.02-0.89)

* Variables of sex, age, education status, family history of esophageal cancer, cigarette smoking, alcohol consumption were adjusted.

Abbreviations: CI, confidence interval; ESCC, esophageal squamous cell carcinoma; HR, hazard ratio; PYs, person-years.

© 2022 Li H et al. *JAMA Network Open.*

eTable 3. Adjusted risk of progression to esophageal squamous cell carcinoma (ESCC) in patients with low-grade intraepithelial neoplasia over a median follow-up period of 7.96 years, adjusted by surveillance endoscopy visits and surveillance interval

Variable	No. of patients of LGIN, n (%)	Person years	No. of ESCC cases	Univariate HR (95% CI)	P value	Multivariable HR (95% CI)	P value
No. of surveillance endoscopy visits							
0	1880 (57.70)	15992.97	113	referent		referent	
1	1079 (33.12)	8378.43	37	0.61 (0.42-0.88)	0.009	0.60 (0.41-0.87)	0.007
2	231 (7.09)	2083.45	14	0.95 (0.55-1.66)	0.87	0.88 (0.50-1.54)	0.66
≥3	68 (2.09)	626.41	6	1.37 (0.60-3.11)	0.45	1.27 (0.56-2.90)	0.57
Surveillance endoscopy according to the recommended interval							
No surveillance	1880 (57.70)	15992.97	113	referent		referent	
Surveillance within the recommended interval	934 (28.67)	15992.97	33	0.66 (0.45-0.97)	0.04	0.62 (0.42-0.92)	0.02
Surveillance outside of the recommended interval	444 (13.63)	10479.61	24	0.81 (0.52-1.26)	0.34	0.81 (0.52-1.26)	0.35

Abbreviations: CI, confidence interval; ESCC, esophageal squamous cell carcinoma; HR, hazard ratio; LGIN, low-grade intraepithelial neoplasia.

© 2022 Li H et al. *JAMA Network Open.*

eTable 4: Sensitivity analysis of the association between surveillance endoscopy and the incidence of ESCC

Models	No. of patients of LGIN, n	Person years	No. of ESCC cases	Multivariable HR (95% CI)	P value
Main analysis	3258	27081.26	170	0.69 (0.50-0.95)	0.02
Multiple imputation	3303	27536.68	171	0.69 (0.62-0.76)	<0.001
Exclusion participants enrolled before 2012	2071	14016.78	78	0.60 (0.38-0.96)	0.03

Abbreviations: CI, confidence interval; ESCC, esophageal squamous cell carcinoma; HR, hazard ratio; LGIN, low-grade intraepithelial neoplasia.

© 2022 Li H et al. *JAMA Network Open.*
