# Supplementary material for: Long-term Incidence Rates of Esophageal Squamous Cell Carcinoma in Chinese Patients With Low-grade Intraepithelial Neoplasia and Association of Surveillance Endoscopy With Incidence
Source: JAMA Netw Open. 2022 Dec 19;5(12):e2247415. doi: 10.1001/jamanetworkopen.2022.47415 (PMC9856485; doi:10.1001/jamanetworkopen.2022.47415)
Supplement: Supplement 2. — Data Sharing Statement [file jamanetwopen-e2247415-s002.pdf]

Data Sharing Statement

Li. Long-term Incidence Rates of Esophageal Squamous Cell Carcinoma in Chinese Patients With Low-Grade Intraepithelial Neoplasia and Association of Surveillance Endoscopy With Incidence. *JAMA Netw Open*. Published December 19, 2022.
doi:10.1001/jamanetworkopen.2022.47415

Data
Data available: Data are available upon reasonable request. Data from the study are available upon request from the corresponding author.
